# A Novel Approach to Image Recoloring for Color Vision Deficiency

**DOI:** 10.3390/s21082740

**Published:** 2021-04-13

**Authors:** George E. Tsekouras, Anastasios Rigos, Stamatis Chatzistamatis, John Tsimikas, Konstantinos Kotis, George Caridakis, Christos-Nikolaos Anagnostopoulos

**Affiliations:** 1 Department of Cultural Technology and Communications, University of the Aegean, 811 00 Mitilini, Greece; a.rigos@aegean.gr (A.R.); stami@aegean.gr (S.C.); kotis@aegean.gr (K.K.); gcari@aegean.gr (G.C.); canag@ct.aegean.gr (C.-N.A.); 2 Department of Statistics and Actuarial-Financial Mathematics, University of the Aegean, 811 00 Mitilini, Greece; tsimikas@aegean.gr

**Keywords:** color vision deficiency, image recoloring, confusion line, chromaticity diagram, fuzzy clustering, differential evolution, color transfer, natural images, art paintings

## Abstract

In this paper, a novel method to modify color images for the protanopia and deuteranopia color vision deficiencies is proposed. The method admits certain criteria, such as preserving image naturalness and color contrast enhancement. Four modules are employed in the process. First, fuzzy clustering-based color segmentation extracts key colors (which are the cluster centers) of the input image. Second, the key colors are mapped onto the CIE 1931 chromaticity diagram. Then, using the concept of confusion line (i.e., loci of colors confused by the color-blind), a sophisticated mechanism translates (i.e., removes) key colors lying on the same confusion line to different confusion lines so that they can be discriminated by the color-blind. In the third module, the key colors are further adapted by optimizing a regularized objective function that combines the aforementioned criteria. Fourth, the recolored image is obtained by color transfer that involves the adapted key colors and the associated fuzzy clusters. Three related methods are compared with the proposed one, using two performance indices, and evaluated by several experiments over 195 natural images and six digitized art paintings. The main outcomes of the comparative analysis are as follows. (a) Quantitative evaluation based on nonparametric statistical analysis is conducted by comparing the proposed method to each one of the other three methods for protanopia and deuteranopia, and for each index. In most of the comparisons, the Bonferroni adjusted *p*-values are <0.015, favoring the superiority of the proposed method. (b) Qualitative evaluation verifies the aesthetic appearance of the recolored images. (c) Subjective evaluation supports the above results.

## 1. Introduction

The human trichromatic color vision originates from the comparison of the rates at which photons are absorbed by three types of photoreceptor cone-cells namely, the L-, M-, and S-cones [1,2]. In practice, the above types of cones define the three channels of the LMS color space, and only as an approximation of their stimulation they correspond to the red, green, and blue colors, respectively. Color vision deficiency (CVD) (or color-blindness) is defined as the human eye’s inability to correctly match and perceive colors affecting at least 8% of males and 0.8% of females [2,3]. It is caused by genetic mutations that lead either to the absence or dysfunctionality of one or two types of cones [2,4,5]. The impact of CVD on human vision is reflected on various physiological levels such as color discrimination, object recognition, color appearance, color naming, etc. [1,6].

There are three categories of CVDs [1,2,3,5]. The most severe and the rarest one is the achromatopsia caused by the absence of two types of cones. The second is the dichromacy, where one type of cones is missing. Dichromacy includes three subcategories: protanopia, deuteranopia, and tritanopia, depending on whether the L-, the M-, or the S-cones are missing, respectively. Protanopia and deuteranopia have similar effects and belong to the so-called red–green color vision deficiency. The third is the anomalous trichromacy, where no cone types are missing but at least one of them is malfunctioning. It also comprises three subcategories namely the protanomaly, deuteranomaly and tritanomaly depending on whether the L-, the M-, and the S-cones are affected, respectively.

This paper considers the defects of protanopia and deuteranopia. Since protanopes and deuteranopes lack one primary type of cone, they match the full-color spectrum using the other two primary types of cones. Thus, they confuse only the colors that can be perceived and discriminated by the missing primary type of cone [3].

**Figure 1:**
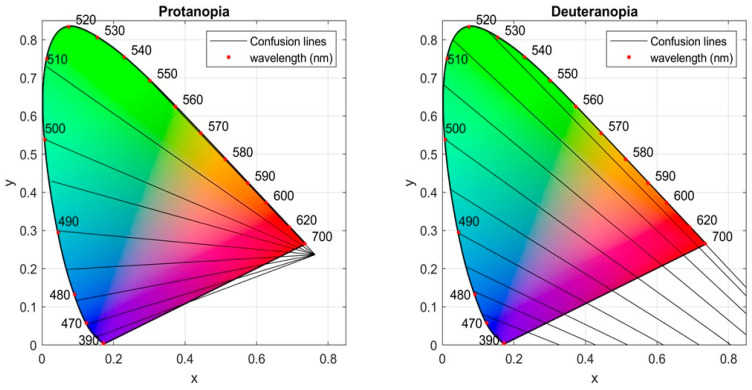
Confusion lines for protanopia (**left diagram**) with copunctal point $\left(x_{cp},\;y_{cp})=(0.763,\;0.236\right)$ and deuteranopia (**right diagram**) with copunctal point $\left(x_{cp},\;y_{cp})=(1.4,\;-0.4\right)$.

An effective tool to describe the above color confusion process is the confusion lines defined on the CIE 1931 chromaticity diagram [4,5]. A confusion line is defined as the locus of the set of colors confused by the protanope or deuteranope. All confusion lines intersect at a point outside the chromaticity diagram, which is called copunctal point and it is imaginary stimulus realized as the locus of the missing primary [3,6]. The color-blind confuses all colors belonging to the same line and therefore the entire line appears achromatic to him [4]. In essence, those colors are perceived by the color-blind with the same colorfulness, which consists of hue and saturation, but they can be identical only under the appropriate intensity [6,7,8]. Figure 1 shows some confusion lines for protanopia and deuteranopia taken from Judd’s revised chromaticity diagram [3,9].

To solve the problem of color-blindness, image recoloring algorithms have been implemented following different approaches. In any case however, the design of image recoloring algorithms must fulfill certain requirements. The most important are the preservation of color naturalness and the preservation or enhancement of color contrast [10]. Naturalness refers to the reduction of the perceptual color difference between the original and the recolored image, and therefore it quantifies their color distribution and aesthetic similarity. Contrast is important for object recognition and color discrimination, especially in the case where two different objects are perceived by the color-blind as one object.

So far, a wide range of recoloring methods have been developed based on optimization of specially designed objective functions [11,12,13,14,15,16] or regularized objective functions [17,18,19] that uniformly combine the naturalness and contrast criteria, pixel-based classification [20], spectral filtering [21], cluster analysis [22], gradient domain recoloring [23,24], confusion-line based [7,8,25], color transformation and rotation/translation [26,27,28,29], neural networks [30], image retrieval [31], and deep learning [32].

In this study, a novel approach to image recoloring for protanopes and deuteranopes is developed. The proposed method encompasses four modules: (a) key color extraction, (b) key color translation, (c) key color optimization, and (d) cluster-to-cluster color transfer. The first module performs color segmentation of the input image using fuzzy clustering to extract a number of cluster centers, called key colors. In the second module, the key colors are mapped in the CIE 1931 chromaticity diagram. Colors confused by the color blind (called here confusing key colors) are ranked in terms of the cardinalities of the associated clusters. Then, an iterative process is set up, where in each iteration the confusing key color with the highest rank is translated (removed) to its closest non-occupied (by other key colors) confusion line. In the third module, only the luminance channel of the translated confusing key colors is further optimized in terms of a regularized objective function that uniformly quantifies the contrast and the naturalness criteria. Finally, the fourth module uses a color transfer mechanism to finalize the recoloring process of the image’s pixels.

The paper is organized as follows. Section 2 presents the state of the art and the current contribution. Section 3 describes the proposed methodology in detail. The experimental evaluation and the respective findings are reported and analyzed in Section 4. Finally, the paper concludes in Section 5.

## 2. State of the Art and the Current Contribution

### 2.1. Image Recoloring for the Color-Blind: State of the Art

One of the most challenging problems in color science is the identification of the dichromatic color appearance so that normal trichromats can realize the way color-blinds experience colors. In their seminal papers, Vienot et al. [33,34] developed an algorithmic framework to simulate the dichromatic color vision. Their findings suggested that protanopia and deuteranopia are reduced forms of normal trichromatic vision. In particular, they showed that the dichromat color gamut is a plane in the three-dimensional RGB space. Having generated the dichromat simulation of an image using the above method, recoloring processes can be applied to carry out color adaptation of the image in order to enhance color appearance, color discrimination, and object recognition for the color-blind [10,13,17].

So far, several approaches have been developed to perform image recoloring. Kuhn et al. [13] transformed the red–green–blue (RGB) dichromatic simulated gamut in the CIELab color space and generated a set of key colors. They minimized an objective function that included distances between key colors and their projections on the Lb plane. Finally, the Lb plane was rotated to match the desired colors. While the method manages to increase the contrast, it finally changes the colors significantly due to the rotation process and the naturalness deteriorates. In [17] the CIELab color space was used to perform image recoloring. Confusing colors were rotated in the ab plane. The rotation angles were calculated by the minimization of a regularized objective function that involved color contrast and naturalness criteria. However, the above minimization might compromise either the contrast or the naturalness requirement. Han et al. [7] also employed the CIELab space, where the original image was segmented into regions and extracted representative colors. Confusing colors belonging to the same confusion line were relocated so that all regions can be discriminated by the color-blind. However, the selected number of confusion lines was very large, exceeding the number of wavelengths seen by the color-blind. Therefore, it is possible colors belonging to two or more confusion lines may still be confused and the contrast of the recolored image is compromised. Huang et al. [25] applied mixture modeling in the CIELab space to partition the colors into clusters. The distance between pairs of cluster centers was generalized to quantify the dissimilarity between pairs of distributions. Based on these dissimilarities an objective function was minimized to obtain an efficient recoloring of the original image. The above method increases the contrast but there is no control over hue changes for the confusing colors and thus, the color naturalness might be compromised. Meng and Tanaka [16] modified the lightness in the CIELab space without changing the hue of the original image. That modification was carried out in terms of an optimization problem where the objective function included color differences. Although this strategy seems to effectively maintain the naturalness, the unchanged hue in combination with the changed lightness may negatively affect the contrast of the recolored image. Kang et al. [15] calculated a set of key colors in the CIELab. The authors estimated the differences between pairs of them and projected the resulting difference vectors on the color subspace seen by the dichromats, which is a plane. Then, they minimized an objective function that included the above projections and attempted to improve the local contrast between color regions of the image, and the image’s global contrast. While the optimization method improves the contrast, there is no mechanism to control the naturalness, which might be compromised.

Apart from the CIELab, a commonly used color space is the Hue-Saturation-Value (HSV). For example, Wong and Bishop [27] showed that effective results can be obtained if the saturation and brightness remain the same as in the original image, while the hue value is shifted by applying a nonlinear mapping that involves a power of the hue value. This process makes confused hue ranges distinguishable from each other. The hues’ remapping process finally improves the contrast, while the power hue shift retains the aesthetics of the original image and thus the naturalness. Similarly, Chin and Sabudin [28] transformed the original image into the HSV color space, where the hue value was appropriately rotated, while the saturation and brightness remained unchanged. Specifically, the ranges of reds and greens are translated towards the ranges of blues and yellows, which are distinguishable by the protanopes and deuteranopes. Using the above strategy, the contrast seems to be enhanced while the naturalness is compromised.

The RGB color space has been considered, also. Ma et al. [30] applied a self-organizing map (SOM) to transform the RGB space into the dichromatic space by preserving the distance ratios between homologous colors. The resulting code-vectors are mapped into a rectangular color palette that has black and white in two opposing corners, blue and yellow in the other two corners, obtaining the final image recoloring. Although the method enhances the color contrast, the naturalness may be compromised because the color palette utilizes only blue and yellow and their variants. In [32], a recoloring for art paintings was proposed. First, a deep learning network was used to perform transfer learning from natural images to art paintings and then a semantic segmentation approach was set up to generate annotated object recognition of art paintings. The recoloring process was carried out by optimizing an objective function that involved only the colors that were significantly different from the respective simulated ones. Since there are recolored only colors associated with the annotated objects the naturalness is improved, but the contrast might be compromised. In [23], the RGB image was represented as a vector-valued function. Then, using the gradient domain, a global transformation followed by a multi-scale reintegration took place to obtain the recolored image. However, it tended to produce visual halo artifacts near strong chromatic edges, which might negatively affect both naturalness and contrast.

Finally, in [26] the original and the simulated images were transformed in the XYZ space, with normalized luminance values. Regarding the other two channels, errors between homologous colors of the original and the simulated images were derived and rotated. As the rotation angle increases, the selected colors are far away from the dichromat plane and vice-versa. In any case, the colors do not change that much for distant colors and the naturalness is preserved. However, the above rotation strategy will not favor contrast enhancement.

### 2.2. The Current Contribution

The method proposed in this paper uses four algorithmic modules (as mentioned in Section 1) and builds on a novel perspective to the image recoloring for the color-blind. To enhance color discrimination and color appearance, the method provides certain contributions, which are delineated as follows:

- The first contribution concerns the number of colors to be modified. In contrast to other approaches that adapt all colors of the input image [26,27,28,30], our approach modifies only the colors confused by the color blind. Since not all image colors are modified it is expected that the recolored image will maintain the naturalness.
- The second contribution assumes that the adaptation of confusing colors should be driven by a confusion-line based approach. Confusion lines are the product of extensive experimentations [3,6,9]. As such, they accurately reflect the way a dichromat perceives colors. In contrast to other approaches that perform the recoloring only in terms of optimization [15,16,23,25,32], this paper introduces a mechanism to remove specific confusing colors to specific confusion lines, thus enhancing the contrast. Since each color is transferred to its closest non-occupied confusion line, it is expected that the naturalness will be preserved, also.
- The third contribution concerns the need to further optimize both naturalness and contrast. Unlike other approaches that use color or plane rotation mechanisms [13,17,27,28], herein we manipulate the luminance channel to minimize a regularized objective that uniformly combines the naturalness and contrast criteria.

In summary, the main idea of the current contribution is to build a four-module approach, where the naturalness and contrast are gradually improved from one module to the next, taking into account specific requirements.

## 3. The Proposed Method

### 3.1. Preliminaries

**Figure 2:**
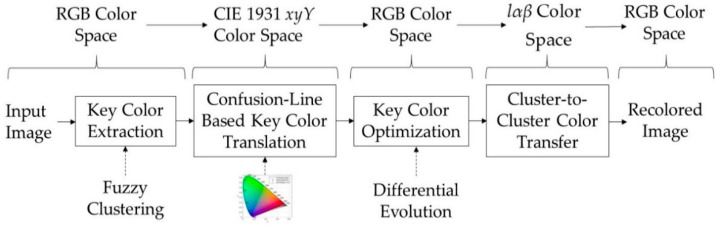
The basic structure of the proposed recoloring method.

The processing of the proposed method is depicted in Figure 2. There are four operational modules involved. (a) Fuzzy clustering [35] is applied to the input image to extract a set of key colors, which are the resulting cluster centers, in the RGB color space. A subset of key colors corresponds to confusing image colors, called here confusing key colors, while the rest of them to non-confusing colors, called here non-confusing key colors. (b) The confusing key colors are ranked in decreasing order according to the cardinalities of the associated clusters. Then, all key colors are mapped on the CIE 1931 chromaticity diagram defined on the *xyY* color space. An iterative process is carried out, where in each iteration the highest ranked confusing key color, if necessary, is translated (moved) to a different confusion line and becomes discriminated by the color-blind. (c) The modified key colors are transformed back to the RGB space. To avoid suboptimal results, an objective function that combines the naturalness and contrast criteria is minimized by the differential evolution algorithm [36]. This module obtains the final recolored key colors. (d) All pixels of the input image that are not discriminated by the color blind along with the above recolored key colors are mapped in the lαβ space [37]. Then, each pixel is recolored by a cluster-to-cluster color transfer approach, which involves the key colors and the associated clusters. Finally, the recolored pixels are mapped into the RGB space, obtaining the recolored image. The color space pipeline used is depicted on the top of Figure 2.

The mapping from *RGB* to *xyY* is denoted as φ:RGB→xyY, and from *RGB* to lαβ as ψ:RGB→lαβ. In the above transformations, all colors were gamma-corrected, and the procedure was carried out in terms of the CIE Standard Illuminant D65 [1,38]. The analytical description for the former can be found in [1,38], while for the latter in [39,40].

The lαβ space is a transformation of the LMS model and was introduced by Ruderman et al. in [37]. The reason for using this space in this paper is that it reduces the correlation between the three channels, enabling the elimination of undesirable cross-channel effects [39,40]. Moreover, its logarithmic-based nature enables the uniform changes in channel intensity to be equally detectable, which is expected to derive an effective pixel-based recoloring.

To obtain the dichromat simulation of a color we use the algorithm developed by Vienot et al. [33,34]. This algorithm shows that the dichromat color space perceived by people suffering from dichromacy is a plane in the *RGB* space, denoted as $RGB_{D}$, where *D* refers to protanopia or deuteranopia, interchangeably. Therefore, $RGB_{D}$ is a proper subspace of the *RGB* space, $RGB_{D}\subset RGB$, which means that $x\in RGB_{D}\Rightarrow x\in RGB$. The mapping, from RGB to $RGB_{D}$ is $f_{D}:\;\;RGB\to RGB_{D}$. Thus,$RGB_{D}=\left\{x:\;x=f_{D}(c),\;\;c\in RGB\right\}$. Since  c∈RGB its follows that it has three coordinates, one for each channel of the *RGB* model: $c={\left[c_{R}\;\;c_{G}\;\;c_{B}\right]}^{T}$. Relationally, x belongs to the $RGB_{D}$ and, as showed above, it also belongs to the *RGB* space. Therefore, x has also three channel values: $x={\left[x_{R}\;\;x_{G}\;\;x_{B}\right]}^{T}$. A detailed description for the calculations of the transformation $f_{D}(\cdot )$ is given in [33,34].

### 3.2. Module 1: Key Color Extraction

The N×M sized input image is $P=\left[p_{t_{1}t_{2}}\right]$ with $1\leq t_{1}\leq N$, $1\leq t_{2}\leq M$ and $p_{t_{1}t_{2}}$ is the $\left(t_{1},\;t_{2}\right)$ pixel of the image. To reduce the computational complexity, the image pixels are grouped into *m* color bins $B_{k}\;\;(1\leq k\leq m)$, with radius ρ. The radius is selected to be neither very small (because the computational time would be increased) nor large (because the resulting color matching would be inefficient). Through experimentation we found that a credible interval is ρ∈[5, 10]. For each bin, the representative color is the mean of all pixels belonging to that bin. The set of the representative colors is $C=\left\{c_{1},\;c_{2},\;\ldots ,\;\;c_{m}\right\}$, with m<N⋅M. The dichromat simulation of *C* is:$f_{D}\left(C\right)=\left\{f_{D}\left(c\right):\;\;c\in C\right\}$.

If the color $c_{k}$ and its dichromat simulation $f_{D}\left(c_{k}\right)$ lie in a sphere with radius δ, which is appropriately selected, then the two colors appear to be the same to a protanope or deuteranope, meaning that the color $c_{k}$ is not confused, otherwise it is confused by the color-blind. Thus, the set *C* is divided into two subsets, $C_{A}$ containing confusing colors and $C_{B}$ containing non-confusing colors,

(1) $$C_{A}=\left\{c\in C:\;\;\left\|c-f_{D}\left(c\right)\right\|\geq \delta \right\}$$

(2) $$C_{B}=C-C_{A}$$

The physical meaning of the parameter δ is to decide whether the distance between $c_{k}$ and its dichromat simulation $f_{D}\left(c_{k}\right)$ is small enough. If this is the case, the two colors belong to the same color region in the *RGB* color space and therefore, the color-blind perceives the color $c_{k}$ correctly. Otherwise, they belong to different color regions, meaning that the color blind confuses the color $c_{k}$ with other colors. The parameter takes values between 0 and 255. However, its value should not be very large or very small. Through extensive experimentation on the Flowers and Fruits data sets, which were taken from the McGill’s calibrated color image database [41] and contain 195 calibrated color images, we found that a credible interval for the parameter δ in Equation (1) is δ∈[20, 30].

Next, the well-known fuzzy c-means [35] is applied separately to the sets $C_{A}$ and $C_{B}$. The target is to partition the elements of the above sets into $n_{A}$ and $n_{B}$ fuzzy clusters, respectively. There are several reasons for choosing the fuzzy c-means algorithm. First, it is not sensitive to random initialization. Second, it involves soft competition between cluster centers, which implies that all clusters have the potential to move and to win data avoiding the creation of underutilized small clusters. Finally, it can generate compact and well separated clusters, thus effectively revealing the underlying data structure.

The sets of the resulting cluster centers are,

(3) $$S_{A}=\left\{a_{1},\;a_{2},\;\ldots ,\;a_{n_{A}}\right\}\;\mathrm{and}\;S_{B}=\left\{b_{1},\;b_{2},\;\ldots ,\;b_{n_{B}}\right\}$$

Only for $C_{A}$, the clusters are denoted as: $A_{1},\;A_{2},\;\ldots ,\;A_{n_{A}}$.

**Definition** **1.** *A color* $c_{k}\;\;(1\leq k\leq m)$ *of the set* C *belongs to the cluster* $A_{i}\;(1\leq i\leq n_{A})$ *if it also belongs to the set* $C_{A}$ *and appears to have its maximum membership degree to that cluster. Then, each pixel of the input image belonging to the corresponding bin* $B_{k}\;\;(1\leq k\leq m)$ *also belongs to the cluster* $A_{i}$ *. The cardinality of the cluster* $A_{i}$ *is defined as the number of pixels of the input image that belong to* $A_{i}$ *, and it is denoted as* $\left|A_{i}\right|$ *.*

The elements of $S_{A}$ are the key colors of the confusing image colors, called confusing key colors, and the elements of $S_{B}$ the key colors of the non-confusing image colors, called non-confusing key colors. The problem is how to appropriately recolor the key colors of $S_{A}$ to obtain the set $S_{A,rec}$

(4) $$S_{A,rec}=\left\{a_{1,rec},\;a_{2,rec},\;\ldots ,\;a_{n_{A},rec}\right\}$$

where *rec* stands for the recoloring. The next two modules (presented in Section 3.3 and Section 3.4) describe in detail the above task.

### 3.3. Module 2: Key Color Translation

The sets $S_{A}$ and $S_{B}$ are mapped onto the *xyY* space as,

(5) $$\varphi \left(S_{A}\right)=\left\{\varphi \left(a_{1}\right),\;\varphi \left(a_{2}\right),\ldots ,\;\varphi \left(a_{n_{A}}\right)\right\}$$

(6) $$\varphi \left(S_{B}\right)=\left\{\varphi \left(b_{1}\right),\;\varphi \left(b_{2}\right),\ldots ,\;\varphi \left(b_{n_{B}}\right)\right\}$$

with

(7) $$\varphi \left(a_{i}\right)={\left[\varphi {\left(a_{i}\right)}_{x},\;\;\varphi {\left(a_{i}\right)}_{y},\;\varphi {\left(a_{i}\right)}_{Y}\right]}^{T},\;i=1,\;2,\;\ldots ,\;n_{A}$$

(8) $$\varphi \left(b_{j}\right)={\left[\varphi {\left(b_{j}\right)}_{x},\;\;\varphi {\left(b_{j}\right)}_{y},\;\varphi {\left(b_{j}\right)}_{Y}\right]}^{T},\;j=1,\;2,\;\ldots ,\;n_{B}$$

where $\varphi {\left(a_{i}\right)}_{x}$ is the hue, $\varphi {\left(a_{i}\right)}_{y}$ the colorfulness, and $\varphi {\left(a_{i}\right)}_{Y}$ the relative luminance coordinate. The latter takes values in [0, 100]. Note that, in view of Equations (4), (5), and (7), the problem is to recolor the elements of $\varphi \left(S_{A}\right)$ and map them back to the RGB space.

The CIE 1931 chromaticity diagram is the projection of the *xyY* color space on the *xy*-plane [1,9,38]. Thus, the key colors in Equations (7), (8) are projected as points on the *xy*-plane,

(9) $$v_{i}={\left[v_{ix},\;\;v_{iy}\right]}^{T}={\left[\varphi {\left(a_{i}\right)}_{x},\;\;\varphi {\left(a_{i}\right)}_{y}\right]}^{T}$$

(10) $$u_{j}={\left[u_{ix},\;\;u_{iy}\right]}^{T}={\left[\varphi {\left(b_{j}\right)}_{x},\;\;\varphi {\left(b_{j}\right)}_{y}\right]}^{T}$$

The above points include only the chromaticity coordinates, which consists of hue and colorfulness. By defining the sets $V=\left\{v_{1},\;v_{2},\;\ldots ,\;v_{n_{A}}\right\}$ and $U=\left\{u_{1},\;u_{2},\;\ldots ,\;u_{n_{B}}\right\}$, we can easily verify that there is a bijective correspondence between *V* and $S_{A}$ (thus, between *V* and $\left\{A_{1},\;A_{2},\;\ldots ,\;A_{n_{A}}\right\}$), and also between *U* and $S_{B}$.

To recolor the elements of $\varphi \left(S_{A}\right)$ we must, first, recolor the elements of *V*. The recoloring of *V* is denoted as $V_{rec}=\left\{v_{1,rec},\;v_{2,rec},\;\ldots ,\;v_{n_{A},rec}\right\}$. To address this issue, a confusion-line based algorithm has been developed, which is described within the next paragraphs.

The colors $\left\{v_{1},\;v_{2},\;\ldots ,\;v_{n_{A}}\right\}$ are ranked in decreasing order according to the cardinalities of the associated clusters $\left\{A_{1},\;A_{2},\;\ldots ,\;A_{n_{A}}\right\}$,

(11) $$rank(v_{i})={\left|A\right|}_{i}/\sum_{\ell =1}^{n_{A}}{\left|A\right|}_{\ell }$$

The reason for using the above ranking function is that a key color corresponding to a cluster with large cardinality is associated with a large image area, and its contribution to the final result should be more important.

Proper subsets of the set of confusion lines of Judd’s revised chromaticity diagram [3,9] for protanopia and deuteranopia are employed. The number of total confusion lines is defined as $Q_{D}$, where *D* refers either to protanopia or deuteranopia. All colors belonging to the same confusion line are not discriminated one from another and they are perceived as a single color by the color-blind. Using the standard point-to-line distance, each one of the colors of *V* and *U* is assigned to its closest confusion line. The distance of the point $v=(v_{x},\;v_{y})$ to the confusion line L that passes through the copunctal point $\left(x_{cp},\;y_{cp}\right)$ and a point $\left(x_{0},\;y_{0}\right)$ in the chromaticity diagram is given as follows,

(12) $$d\left(v,\;L\right)=\frac{\left|\left(x_{cp}-x_{0}\right)\left(y_{0}-v_{y}\right)-\left(x_{0}-v_{x}\right)\left(y_{cp}-y_{0}\right)\right|}{\sqrt{{\left(x_{cp}-x_{0}\right)}^{2}+{\left(y_{cp}-y_{0}\right)}^{2}}}$$

Each confusion line is labeled as “occupied” if it contains at least one of the colors $\left\{v_{1},\;v_{2},\;\ldots ,\;v_{n_{A}}\right\}$ and $\left\{u_{1},\;u_{2},\;\ldots ,\;u_{n_{B}}\right\}$, or “non-occupied” if it does not contain any colors. The set of the “non-occupied” confusion lines is $CL_{D}=\left\{L_{1},\;L_{2},\;\ldots ,\;L_{\left|CL_{D}\right|}\right\}$, where |⋅| stands for set cardinality.

Next, we identify colors of the set *V* to be translated to different confusion lines. Regarding the occupied confusion lines, the following cases can happen:

- Case 1: A confusion line contains at least one color from the set *U*. If it also contains colors from the set *V*, then all these colors are going to be translated to separate confusion lines.
- Case 2: A confusion line does not contain colors from the set *U*, but it contains at least two colors from the set *V*. In this case, the color with the lowest rank remains on the confusion line, while the rest of the colors are translated to different confusion lines.
- Case 3: A confusion line contains only one color, which belongs to the set *V*. In this case, no color is going to be translated.

The identified colors to be translated form the set $\Phi_{V}$, with $\Phi_{V}\subseteq V$. The point-to-line distances (see Equation (12)) between colors belonging to $\Phi_{V}$, which are points on the chromaticity diagram, and lines belonging to the set $CL_{D}$ are calculated. In addition, the standard projection of the point v on the line L is calculated and symbolized as proj(v, L).

Then, an iterative algorithm takes place. Each iteration involves the following steps.

First the algorithm identifies the color $v_{*}\in \Phi_{V}$ with the highest rank as,

(13) $$v_{*}=\left\{v’\in \Phi_{V}:\;\;rank(v’)=\underset{v\in \Phi_{V}}{\max}\left\{rank(v)\right\}\right\}$$

Then, the non-occupied confusion line $L_{*}\in CL_{D}$ that is closer to $v_{*}$ is determined,

(14) $$d(v_{*},\;L_{*})=\underset{L\in CL_{D}}{\min}\left\{d(v_{*},\;L)\right\}$$

Next, the $v_{*}$ is translated to its projection point $v_{*,tr}$ on the line $L_{*}$,

(15) $$v_{*,tr}=proj\left(v_{*},\;L_{*}\right)$$

Since the color $v_{*}$ has been removed it must be deleted form the set $\Phi_{V}$, while the same holds for the line $L_{*}$ and the set $CL_{D}$. Thus, the iteration ends with the updating process of the sets $\Phi_{V}$ and $CL_{D}$,

(16) $$\Phi_{V}=\Phi_{V}-\left\{v*\right\}$$

(17) $$CL_{D}=CL_{D}-\left\{L*\right\}$$

Using the above iterative process, all colors of $V=\left\{v_{1},\;v_{2},\;\ldots ,\;v_{n_{A}}\right\}$ are recolored,

(18) $$v_{i,rec}=\left\{ \begin{array}{l} v_{i,tr},\;\;\;\;if\;\;v_{i}\;\;was\;\;translated\;\\ \;\;\;\;\;\;v_{i}\;,\;\;\;\;otherwise \end{array}\right.$$

Finally, we obtain the recoloring of the set V as follows,

(19) $$V_{rec}=\left\{v_{1,rec},\;v_{2,rec},\;\ldots ,\;v_{n_{A},rec}\right\}$$

The next algorithm presents the above steps in a systematic manner.
**Algorithm 1: Translation process of the colors belonging to the set *V*****Inputs**: The sets V, $\Phi_{V}$, $CL_{D}$; **Output**: The set $V_{rec}$Set ${\left|\Phi_{V}\right|}_{0}=\left|\Phi_{V}\right|$ and ${\left|CL_{D}\right|}_{0}=\left|CL_{D}\right|$**While**$\left|\Phi_{V}\right|>0$**and**$\left|CL_{D}\right|>0$**do** 1.Apply Equation (13) to estimate the $v_{*}\in \Phi_{V}$ with the highest rank. 2.Apply Equation (14) to identify the confusion line $L_{*}\in CL_{D}$, which is closer to $v_{*}$. 3.Apply Equation (15) to translate the color $v_{*}$ to its projection point $v_{*,tr}$ lying on $L_{*}$. 4.Apply Equation (16) to update the set $\Phi_{V}$ 5.Apply Equation (17) to update the set $CL_{D}$**End While** 6.Apply Equation (18) to recolor the elements of V and form the recolored set $V_{rec}$ in Equation (19)

**Remark** **1**. *Regarding Algorithm 1, the following observations are brought into spotlight.*

*1.*  *It is possible that at least two colors will move to distant confusion lines. Although this will increase contrast, the naturalness will be compromised.*
*2.*  *It is recommended* $n_{A}+n_{B}\leq Q_{D}$ *so that* $\left|\Phi_{V}\right|\leq \left|CL_{D}\right|$ *, and all colors of* $\Phi_{V}$ *will move to different confusion lines. We performed extensive experiments on the Flowers and Fruits data sets, which contain 195 calibrated color images and were taken from the McGill’s calibrated color image database [41] and found that the above condition is effective as far as the color segmentation of the input image is concerned. However, depending on the designer’s choice, if* $n_{A}+n_{B}>Q_{D}$ *it is possible to get* $\left|\Phi_{V}\right|>\left|CL_{D}\right|$ *and some key colors of* $\Phi_{V}$ *will not be removed. In this case the naturalness will be enhanced, and the contrast will be reduced.*

**Remark** **2.** *In steps 1–3 the key color with the highest rank is translated first, while the key color with the lowest rank last. Given that the higher ranked key color corresponds to larger image area, the reasons behind this choice are enumerated as follows:*

*1.*  *Let us assume that there is an occupied confusion line, which falls in the above-mentioned Case 1. Thus, the confusion line contains key colors from the sets U and V and therefore, all key colors belonging to V and lying on that confusion line must be translated. By translating, first, the key color with the highest rank, this color will be removed to its closest non-occupied confusion line, and the final color will be close to the original one. In this direction, a low ranked key color will be removed to a distant non-occupied confusion line. Following this strategy, large image areas will be recolored using colors similar to the original ones, while small image areas using colors much different to the original ones. This fact directly implies that the recolored image will preserve the naturalness criterion. On the other hand, if we choose to remove the low ranked key colors first, the opposite effect will take place and the naturalness criterion of the recolored image will be seriously compromised.*
*2.*  *Let us assume that there is an occupied confusion line, which falls in the above-mentioned Case 2. Thus, the confusion line contains key colors from the set V and therefore all but one key colors must be translated. If we choose to remove the low ranked key colors first, then the non-occupied confusion lines closer to the above occupied one will be exhausted, and the higher ranked key colors will be forced to be removed to distant confusion lines. Thus, large images areas will be recolored using much different colors to the original one and the naturalness will be seriously damaged. Yet, the highest ranked key color will remain the same. However, there is no guarantee that this counterbalancing effect will be strong enough to improve the naturalness criterion.*

**Figure 3:**
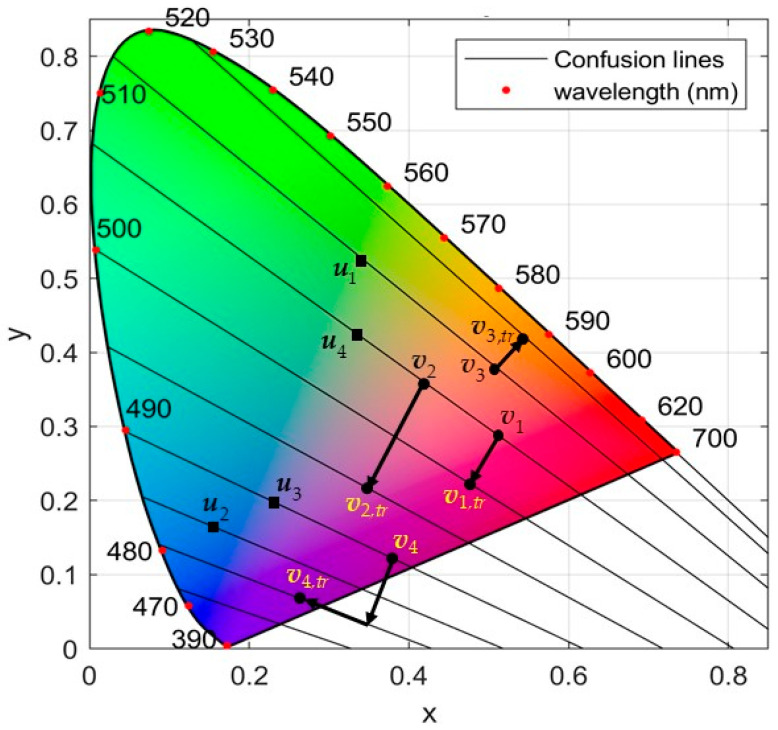
The key color translation process, where $rank(v_{3})>rank(v_{1})>rank(v_{2})>rank(v_{4})$.

Figure 3 illustrates the mechanism of Algorithm 1 using four color translations. Note that the projection of color $v_{4}$ lies outside the chromaticity diagram. This is a rare situation, and the final position of $v_{4,tr}$ is selected above the diagram’s baseline, called “line of purples”. This line contains non-spectral colors. Therefore, the selected point is located at a very small distance ε=0.01 above that line. Since we are moving on the same confusion line, the result will be the same for the color-blind.

So far, the translation process focused on the chromaticity values $\varphi {\left(a_{i}\right)}_{x}$ and $\varphi {\left(a_{i}\right)}_{y}$ of the color $\varphi \left(a_{i}\right)$ (see Equations (7) and (9)). Thus, the luminance coordinates $\varphi {\left(a_{i}\right)}_{Y}$ $\left(i=1,\;2,\;\ldots ,\;n_{A}\right)$ have not been considered yet. A simple approach to this problem would be to avoid changing the values of luminance. However, in view of the difficulties reported in Remarks 1 and 2, keeping the input image’s luminosity might lead to suboptimal results as far as the naturalness and contrast criteria are concerned. Therefore, we further optimize the adapted key colors by manipulating their luminance values only. This task will be presented in the next module. For the moment, we complete the analysis of the current module as indicated in the next paragraphs. Based on Equations (9) and (18) it follows that,

(20) $$v_{i,rec}={\left[v_{ix,rec},\;\;v_{iy,rec}\right]}^{T}={\left[\varphi {\left(a_{i}\right)}_{x,rec},\;\;\varphi {\left(a_{i}\right)}_{y,rec}\right]}^{T}$$

Using Equations (5), (7), (18), and (20) the recoloring of $\varphi \left(S_{A}\right)$ reads as,

(21) $$\varphi {\left(S_{A}\right)}_{rec}=\left\{\varphi {\left(a_{1}\right)}_{rec},\;\varphi {\left(a_{2,\;rec}\right)}_{rec},\ldots ,\;\varphi {\left(a_{n_{A}}\right)}_{rec}\right\}$$

where

(22) $$\varphi {\left(a_{i}\right)}_{rec}={\left[\varphi {\left(a_{i}\right)}_{x,rec},\;\;\varphi {\left(a_{i}\right)}_{y,rec},\;\varphi {\left(a_{i}\right)}_{Y,rec}\right]}^{T}$$

Note that the luminance values of the recolored key colors are defined as $\varphi {\left(a_{i}\right)}_{Y,rec}$ $\left(i=1,\;2,\;\ldots ,\;n_{A}\right)$. Their values will be determined in the next section in terms of an optimization procedure.

### 3.4. Module 3: Key Color Optimization

Having estimated the recolored chromaticity values $\varphi {\left(a_{i}\right)}_{x,rec}$ and $\varphi {\left(a_{i}\right)}_{y,rec}$ for the key colors of the set $\varphi {\left(S_{A}\right)}_{rec}$, the respective luminance values $\varphi {\left(a_{i}\right)}_{Y,rec}$ should be defined as functions of the input image’s luminance values $\varphi {\left(a_{i}\right)}_{Y}$. If $\varphi {\left(a_{i}\right)}_{Y,rec}>>\varphi {\left(a_{i}\right)}_{Y}$ then the image will be much brighter, while in the opposite case much darker. Imposing such aggressive changes in the input image’s luminance will negatively affect the balance between naturalness and contrast. To resolve this problem, the following domain of values for the recolored luminance values $\varphi {\left(a_{i}\right)}_{Y,rec}\in \Theta_{i}$ is suggested,

(23) $$\Theta_{i}=\left\{ \begin{array}{l} \;\;\;\;\;\;\;\;\;\;\;\;\;\left(0,\;\;\varphi {\left(a_{i}\right)}_{Y}+\gamma \right]\;\;\;\;\;\;\;\;\;\;,\;\;if\;\;\varphi {\left(a_{i}\right)}_{Y}<\gamma \\ \;\;\;\;\;\;\;\;\;\left[\varphi {\left(a_{i}\right)}_{Y}-\gamma ,\;\;100\right]\;\;\;\;\;\;\;\;\;\;,\;\;if\;\;100-\varphi {\left(a_{i}\right)}_{Y}<\gamma \\ \left[\varphi {\left(a_{i}\right)}_{Y}-\gamma ,\;\;\varphi {\left(a_{i}\right)}_{Y}+\gamma \right]\;\;,\;\;otherwise \end{array}\right.$$

In general, the above relation implies that the recolored luminance $Y_{rec}$ lies within an interval with radius equal to γ and center the original image luminance *Y*, with $Y_{rec}\in \left[Y-\gamma ,\;\;Y+\gamma \right]$, retaining the edges of the interval [0, 100] inviolable. The parameter γ should neither be large nor small. Through trial-and-error it was found that a credible value is γ=5.

The set $\varphi {\left(S_{A}\right)}_{rec}$ is mapped from the *xyY* into the *RGB* space as $S_{A,rec}=\varphi^{-1}\left(\varphi {\left(S_{A}\right)}_{rec}\right)$, where based on Equations (4), (20), and (21) each element of $S_{A,rec}$ is,

(24) $$a_{i,rec}=\varphi^{-1}\left(\varphi {\left(a_{i}\right)}_{rec}\right)=h\left(\varphi {\left(a_{i}\right)}_{Y,rec}\right),\;\left(i=1,\;2,\;\ldots ,\;n_{A}\right)$$

Note that the above inverse mapping defines the $a_{i,rec}\in RGB$ as a function of the corresponding luminance $\varphi {\left(a_{i}\right)}_{Y,rec}$. The dichromat simulation of $S_{A,rec}$ is,

(25) $$f_{D}\left(S_{A,rec}\right)=\left\{f_{D}(a_{1,rec}),\;f_{D}(a_{2,rec}),\;\ldots ,\;f_{D}(a_{n_{A},rec})\right\}$$

To this end, there are five sets involved in the optimization approach namely, $S_{A}$ given in Equation (3), $S_{A,rec}$ reported in Equation (4) and its dichromat simulation $f_{D}\left(S_{A,rec}\right)$ in Equation (25), $S_{B}$ in Equation (3) and its dichromat simulation $f_{D}\left(S_{B}\right)=\left\{f_{D}\left(b_{1}\right),\;f_{D}\left(b_{2}\right),\;\ldots ,\;f_{D}\left(b_{n_{B}}\right)\right\}$. Recalling that $S_{A}$ and $S_{B}$ respectively include the key colors that correspond to the clusters of image’s confusing and non-confusing colors, the color differences between the elements of $S_{A}$ and $S_{B}$ as perceived by a normal trichromat are determined as $\left\|a_{i}-b_{j}\right\|\;\;\;\left(1\leq i\leq n_{A};\;1\leq j\leq n_{B}\right)$. After the recoloring, the same differences as seen by a dichromat color-blind are defined between the elements of the sets $f_{D}\left(S_{A,rec}\right)$ and $f_{D}\left(S_{B}\right)$ as $\left\|f_{D}\left(a_{i,rec}\right)-f_{D}\left(b_{j}\right)\right\|$. To enable a dichromat to perceive the color differences in a similar way as a normal trichromat does, thus enhancing the contrast of the recolored image, the above distances must be as similar as possible [32],

(26) $$E_{1}=\frac{1}{n_{A}n_{B}}\sum_{i=1}^{n_{A}}\sum_{j=1}^{n_{B}}\left|\left\|a_{i}-b_{j}\right\|-\left\|f_{D}\left(a_{i,rec}\right)-f_{D}\left(b_{j}\right)\right\|\right|$$

Following the same reasoning between the elements of $S_{A}$ and $f_{D}\left(S_{A,rec}\right)$ [32],

(27) $$E_{2}=\frac{1}{n_{A}n_{B}}\sum_{i=1}^{n_{A}}\sum_{j=1}^{n_{B}}\left|\left\|a_{i}-a_{j}\right\|-\left\|f_{D}\left(a_{i,rec}\right)-f_{D}\left(a_{j,rec}\right)\right\|\right|$$

Thus, $E_{1}$ and $E_{2}$ quantify the contrast enhancement of the recolored image [32]. To further improve the naturalness, the subsequent error function is employed [32],

(28) $$E_{3}=\frac{1}{n_{A}}\sum_{i=1}^{n_{A}}\left\|a_{i}-a_{i,rec}\right\|$$

Based on the above analysis, the overall optimization approach is defined as,

(29) $$\mathrm{Minimize}\text{:}\;E=\left(E_{1}+E_{2}\right)+\lambda E_{3}$$

with respect to the luminance variables $\varphi {\left(a_{i}\right)}_{Y,rec}\;\;\;\;i=1,\;2,\;\ldots ,\;n_{A}$.

As indicated in Equation (23) $\varphi {\left(a_{i}\right)}_{Y,rec}\in \Theta_{i}$. Note that Equation (24) defines the colors $a_{i,rec}$ as functions of $\varphi {\left(a_{i}\right)}_{Y,rec}$. The parameter λ is the regularization factor that takes positive values, and its functionality relies on obtaining a counterbalance between contrast and naturalness.

To perform the above optimization the well-known differential evolution (DE) algorithm is applied [36]. The DE is carried out in three evolutionary phases namely, mutation, crossover, and selection. It also includes two pre-defined learning parameters: (a) the parameter that controls the population’s evolving rate denoted as $F_{R}\in \left(0,\;\;1\right]$, and (b) the parameter that controls the fraction of the feature values copied from one generation to the next, denoted as $C_{R}\in \left[0,\;\;1\right]$. The population consists of q particles. Each particle encodes the luminance variables, $\varphi {\left(a_{1}\right)}_{Y,rec},\;\;\;\varphi {\left(a_{2}\right)}_{Y,rec},\;\;\ldots ,\;\;\;\varphi {\left(a_{n_{A}}\right)}_{Y,rec}$. Thus, the size of feature space where the DE searches for a solution is $n_{A}$. The maximum number of iterations is $t_{\max}$.

The result of the above-mentioned optimization is the final calculation of the recoloring set $S_{A,rec}=\left\{a_{1,rec},\;a_{2,rec},\;\ldots ,\;a_{n_{A},rec}\right\}$.

### 3.5. Module 4: Cluster-to-Cluster Color Transfer

The implementation of this module obtains the recolored image, eventually. The key idea is to elaborate on the clusters $A_{1},\;A_{2},\;\ldots ,\;A_{n_{A}}$, the respective cluster centers $S_{A}=\left\{a_{1},\;a_{2},\;\ldots ,\;a_{n_{A}}\right\}$, and the recolored centers $S_{A,rec}=\left\{a_{1,rec},\;a_{2,rec},\;\ldots ,\;a_{n_{A},rec}\right\}$, obtained in the previous module, with $a_{i}\in RGB$ and $a_{i,rec}\in RGB$.

According to Definition 1, each cluster $A_{i}$ includes $\left|A_{i}\right|$ pixels from the input image, which correspond to confusing colors. Those pixels along with the cluster center $a_{i}$ and its recoloring $a_{i,rec}$ are mapped from *RGB* in the lαβ space as

(30) $$p_{t_{1}t_{2}}^{l\alpha \beta }=\psi \left(p_{t_{1}t_{2}}\right)={\left[p_{t_{1}t_{2}}^{l}\;\;p_{t_{1}t_{2}}^{\alpha }\;\;p_{t_{1}t_{2}}^{\beta }\right]}^{T}\;\mathrm{such}\;\mathrm{that}\;p_{t_{1}t_{2}}\in A_{i}$$

(31) $$a_{i}^{l\alpha \beta }=\psi \left(a_{i}\right)={\left[a_{i}^{l}\;\;a_{i}^{\alpha }\;\;a_{i}^{\beta }\right]}^{T}$$

and

(32) $$a_{i,rec}^{l\alpha \beta }=\psi \left(a_{i,rec}\right)={\left[a_{i,rec}^{l}\;\;a_{i,rec}^{\alpha }\;\;a_{i,rec}^{\beta }\right]}^{T}$$

In [39,40], Reinhard et al. developed a color transfer technique between images. In this paper, we adopt that technique and use it to perform color transfer between clusters. A detailed analysis of the color transfer technique is given in [39,40].

Our target is to obtain a recoloring $p_{t_{1}t_{2},rec}^{l\alpha \beta }={\left[p_{t_{1}t_{2},rec}^{l}\;\;p_{t_{1}t_{2},rec}^{\alpha }\;\;p_{t_{1}t_{2},rec}^{\beta }\right]}^{T}$ of the pixels $p_{t_{1}t_{2}}^{l\alpha \beta }$ that belong to the cluster $A_{i}$. We can easily calculate the standard deviation vector $\sigma_{i}={\left[\sigma_{i}^{l}\;\;\;\sigma_{i}^{\alpha }\;\;\sigma_{i}^{\beta }\right]}^{T}$ of the cluster $A_{i}$ in lαβ color space.

Upon the assumption that $a_{i,rec}^{l\alpha \beta }$ is the center of a hypothetical cluster, which has the same cardinality with $A_{i}$ and the same standard deviation $\sigma_{i,rec}=\sigma_{i}$, we use the mechanism of Reinhard et al. [39,40] to transfer the color of the second hypothetical cluster to the first cluster and to obtain the recoloring of each pixel as follows,

(33) $$p_{t_{1}t_{2},rec}^{l\alpha \beta }=\left[\begin{array}{l} p_{t_{1}t_{2},rec}^{l}\\ p_{t_{1}t_{2},rec}^{\alpha }\\ p_{t_{1}t_{2},rec}^{\beta } \end{array}\right]=\left(\left[\begin{array}{l} p_{t_{1}t_{2}}^{l}\\ p_{t_{1}t_{2}}^{\alpha }\\ p_{t_{1}t_{2}}^{\beta } \end{array}\right]-\left[\begin{array}{l} a_{i}^{l}\\ a_{i}^{\alpha }\\ a_{i}^{\beta } \end{array}\right]\right)+\left[\begin{array}{l} a_{i,rec}^{l}\\ a_{i,rec}^{\alpha }\\ a_{i,rec}^{\beta } \end{array}\right]$$

The colors $p_{t_{1}t_{2},rec}^{l\alpha \beta }$ are mapped back to the *RGB* space as,

(34) $$p_{t_{1}t_{2},rec}=\psi^{-1}\left(p_{t_{1}t_{2},rec}^{l\alpha \beta }\right)\;\mathrm{such}\;\mathrm{that}\;p_{t_{1}t_{2}}\in A_{i}$$

Finally, the recolored image $I_{rec}$ is generated by recoloring all pixels belonging to the clusters $A_{1},\;A_{2},\;\ldots ,\;A_{n_{A}}$, while the rest of the pixels remain unchanged.

### 3.6. Computational Complexity Analysis

In this section, the computational complexity of the algorithm is evaluated in terms of distance calculations per iteration.

First, Module 1 is considered. Recalling that the size of the input image is N×M, the number of distance calculations involved in the generation of the bins $B_{k}\;\;(1\leq k\leq m)$ is less than NMm. Also, it is easily verified that for the implementation of Equations (1) and (2), NM distance calculations are needed. On the other hand, it is well known that the complexity of the fuzzy c-means for *c* clusters and H data is $O\left(H\;c^{2}\right)$ [35]. As indicated previously by Equation (3) and the corresponding analysis, the sets $C_{A}$ and $C_{B}$, defined in Equations (1) and (2), are separately clustered by the fuzzy c-means into $n_{A}$ and $n_{B}$ fuzzy clusters, respectively. Thus, the computational complexity of this procedure is $O\left(\left|C_{A}\right|n_{A}^{2}+\left|C_{B}\right|n_{B}^{2}\right)$. By setting $n=\max\left\{n_{A},n_{B}\right\}$, and taking into account that $\left|C_{A}\right|,\;\left|C_{B}\right|\;<m$ it follows that $O\left(\left|C_{A}\right|n_{A}^{2}+\left|C_{B}\right|n_{B}^{2}\right)∼O\left(mn^{2}\right)$. In total, for Module 1 we get $O\left(NMm+Nm+mn^{2}\right)∼O\left(NMm+mn^{2}\right)$. By considering the maximum of $n^{2}$ and NM it follows that $NMm+mn^{2}\leq 2m\;\max\left\{NM,\;n^{2}\right\}$. Thus, the computational complexity of Module 1 is $O\left(m\;\;\max\left\{NM,\;n^{2}\right\}\right)$.

In Module 2, the mappings and the ranking process take place only once, and their effects are not significant. Thus, there are only two dominant effects. The first is related to the implementation of Equation (12) and the second to the implementation of Algorithm 1. The Equation (12) is applied between the elements of the sets *V* and *U* and the $Q_{D}$ confusion lines. The number of distance calculations is $\left(n_{A}+n_{B}\right)Q_{D}<2nQ_{D}$. In Algorithm 1, step 1 involves less than ${\left|\Phi_{V}\right|}_{0}$ calculations, while step 2 less than ${\left|\Phi_{V}\right|}_{0}\;{\left|CL_{D}\right|}_{0}$. Since ${\left|\Phi_{V}\right|}_{0}\leq n_{A}\leq n$ and $\;{\left|CL_{D}\right|}_{0}<Q_{D}$, the number of calculations for Algorithm 1 is less than $\;n(Q_{D}+1)$. Thus, the total number of calculations in Module 2 is less than $2nQ_{D}+n(Q_{D}+1)$, and since $\;Q_{D}$ is fixed it follows that the computational complexity of Module 2 is O(n) .

In Module 3, the estimations of $E_{1},\;E_{2}$, and $E_{3}$ (see Equations (26), (27), and (28)) involve $3n_{A}n_{B}$, $3n_{A}n_{B}$, and $n_{A}$ distance calculations, respectively. It can be easily shown that the sum of the above numbers is less than $6n^{2}+n$. In addition, the implementation of the differential evolution is carried out using *q* particles. Thus, the total number of distance calculations performed in this module is less than $q(6n^{2}+n)$. Since *q* is fixed, the computational complexity of Module 3 is $O\left(n^{2}\right)$.

Regarding Module 4, the most dominant effect is the implementation of Equation (33). The resulting number of calculations is $\sum_{i=1}^{n_{A}}\left|A_{i}\right|<NM$, and the computational complexity of Module 4 is O(NM) .

Thus, the overall computational complexity is $O\left(m\;\;\max\left\{NM,\;n^{2}\right\}+n+n^{2}+NM\right)$. We can easily show that $m\;\;\max\left\{NM,\;n^{2}\right\}+n+n^{2}+NM<4m\;\;\max\left\{NM,\;n^{2}\right\}$, which implies that the computational complexity is modified as $O\left(m\;\;\max\left\{NM,\;n^{2}\right\}\right)$.

## 4. Experimental Evaluation

The performance of the proposed method was evaluated and compared to the respective performances of three related methods. The first, called here Method 1, is the algorithmic scheme developed by Huang et al. in [25]. The second, called here Method 2, was introduced in [27] by Wong and Bishop. Finally, the third, called here Method 3, was developed by Ching and Sabudin in [28]. The basic structures of the three methods are described in Section 2. At this point, it is emphasized that Method 2 produces only one recolored image for both protanopia and deuteranopia [27]. The only difference is that the dichromat simulated images of that recolored image are obviously different for protanopia and deuteranopia. Therefore, the quantitative results obtained by this method are the same for both the protanopia and deuteranopia cases. Table 1 depicts the parameter setting for the proposed method. For the other three methods the parameter settings were the same as reported in the respective referenced papers.

**Figure 4:**
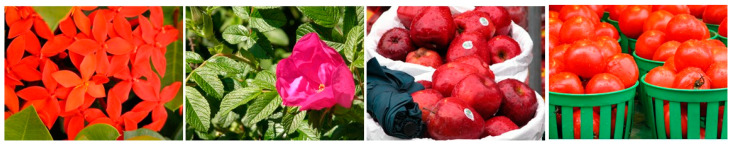
A sample of four images included in the Flowers and Fruits data sets taken from the McGill’s calibrated color image database.

Two data sets were used. The first data set includes the Flowers and Fruits data sets taken from the McGill’s calibrated color image database (http://tabby.vision.mcgill.ca (accessed on 5 January 2021)) [41]. The Flowers data set includes 143 natural images, while the Fruits data set 52 natural images. In total there were 195 natural images. Figure 4 illustrates four of those images.

**Figure 5:**
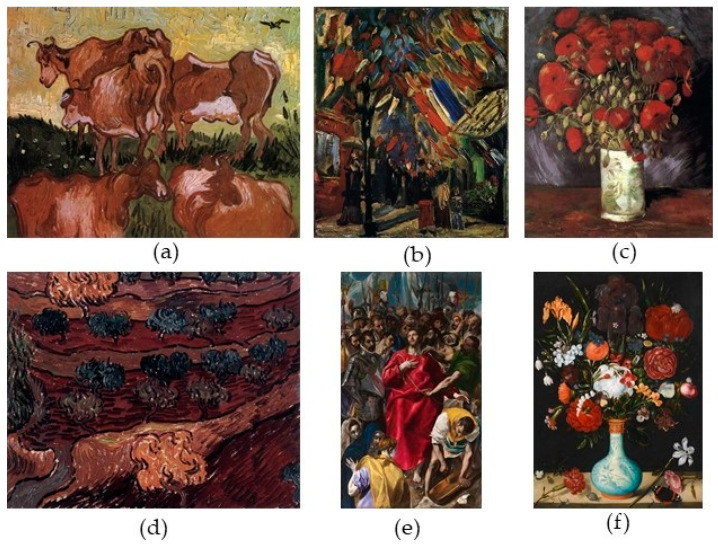
(**a**) Painting 1, (**b**) Painting 2, (**c**) Painting 3, (**d**) Painting 4, (**e**) Painting 5, and (**f**) Painting 6. Paintings 1–4 were created by Vincent van Gogh, Painting 5 by El Greco, and Painting 6 by Ambrosius Bosschaert.

The second data set includes six paintings taken from the Web Gallery of Art (https://www.wga.hu (accessed on 28 September 2020)), which are depicted in Figure 5.

To perform the overall comparison, three kinds of experimental evaluations were conducted, which are analytically described in the following subsections.

### 4.1. Quantitative Evaluation

To conduct the comparative quantitative evaluation of the four methods, two performance indices were employed. First the naturalness index [17,22,32]

(35) $$\mathrm{Jnat}=\frac{1}{N\;M}\sum_{t_{1}=1}^{N}\sum_{t_{2}=1}^{M}\left\|p_{t_{1}t_{2}}-p_{t_{1}t_{2},\;rec}\right\|$$

Second, the feature similarity index (FSIMc) that was developed in [42]. FSIMc takes values in [0, 1] and quantifies the chrominance information of the recolored image in relation to the original image. A higher value of FSIMc indicates that the recolored image’s chrominance information is closer to the chrominance information of the original image. The interested reader may refer to [42] for a detailed description of the index, while a brief derivation of its basic structure is given in the Appendix A.

#### 4.1.1. Quantitative Evaluation Using the Data Set of the Art Paintings

In this evaluation, the six art paintings are used to compare the four methods. Specifically, the values of the two indices Jnat and FSIMc obtained by the four methods are directly compared. In addition, diagrammatic illustration of the translation process is given.

Table 2 presents the results for the naturalness index. Apart from the simulations in Painting 6 (concerning both protanopia and deuteranopia), the proposed method outperforms the other three methods, indicating a high-quality natural appearance of the recolored images when they are viewed by a normal trichromat. This fact is also supported by the subjective evaluation presented later on in this paper.

Table 3 reports the FSIMc index values obtained by the four methods for both dichromacy cases. This table shows some interesting results. Regarding the protanopia case, it seems that Method 1 appears to be very competitive to our proposed method. Similar results are reported in the case of deuteranopia, where, in addition to the previous results, Method 3 gives the best index value for Painting 1. In total, the proposed method achieves a competitive performance when compared to the other methods, which directly implies that the chrominance information of the recolored paintings is close to the original ones.

**Figure 6:**
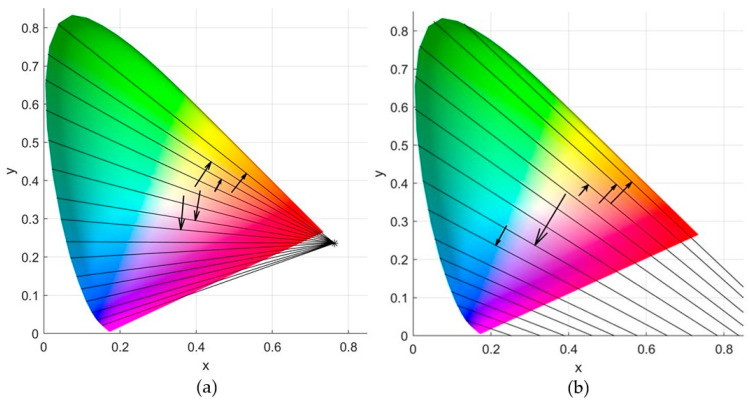
Translation process (see Algorithm 1) for the confusing key colors: (**a**) Painting 1 for the protanopia case; (**b**) Painting 5 for the deuteranopia case.

Figure 6 depicts a sample of two key color translations as described in Algorithm 1. The first refers to Painting 1 and concerns the protanopia case, while the second refers to Painting 5 and concerns the deuteranopia case.

#### 4.1.2. Quantitative Evaluation Using the Data Set of Natural Images

Herein, the four algorithms are compared in terms of statistical analysis using the two above-mentioned indices and considering the 195 calibrated natural images taken McGill’s color image database [41].

For both protanopia and deuteranopia we explored differences between the four methods separately for the Jnat index and the FSIMc index. We utilized nonparametric methods for statistical inference. For each CVD type by index combination, we employed an overall 0.05 significance level. The Friedman test was used to assess differences in median between the four methods. Follow-up, pairwise comparisons between our proposed method and the other three were conducted using the Wilcoxon signed rank procedure. The confidence intervals and associated *p*-values concerning follow-up comparisons reported in this paper are Bonferroni adjusted to ensure an overall 0.05 type-I error for each CVD type by index combination. A detailed report of the results of the statistical analysis follows, separately for each CVD type.

**Figure 7:**
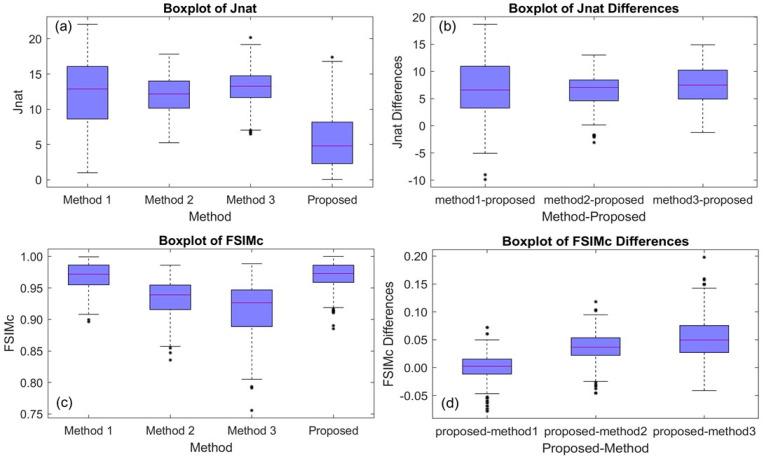
Boxplots for protanopia case considering the McGill’s Flowers and Fruits data sets (in total 195 images): (**a**) the Jnat values (see Table 4), (**b**) Jnat differences between the three competing methods and the proposed method (see Table 5), (**c**) FSIMc values (see Table 4), and (**d**) FSIMc differences between the three competing methods and the proposed method (see Table 5).

Table 4 and Figure 7a summarize the Jnat results for Protanopia. There were statistically significant differences in median Jnat between the four methods. The overall *p*-value was <0.001 (based on the Friedman test).

Table 5 and Figure 7b provide a summary of the differences in Jnat (competing method value–proposed method value). The median difference was statistically significantly greater than zero. The *p*-values are Bonferroni adjusted and based on the Wilcoxon signed rank testing procedure. We conclude that our proposed method was superior to the other three.

Table 4 and Figure 7c summarize the FSIMc results for protanopia. There were statistically significant differences in median FSIMc between the four methods (*p*-value < 0.001).

Table 5 and Figure 7d provide a summary of the differences in FSIMc (proposed method value–competing method value). The *p*-values are Bonferroni adjusted and based on the Wilcoxon signed rank testing procedure. Our proposed method was superior to Method 2 and Method 3. On the other hand, the median difference in FSIMc between our method and Method 1 was not statistically significant.

**Figure 8:**
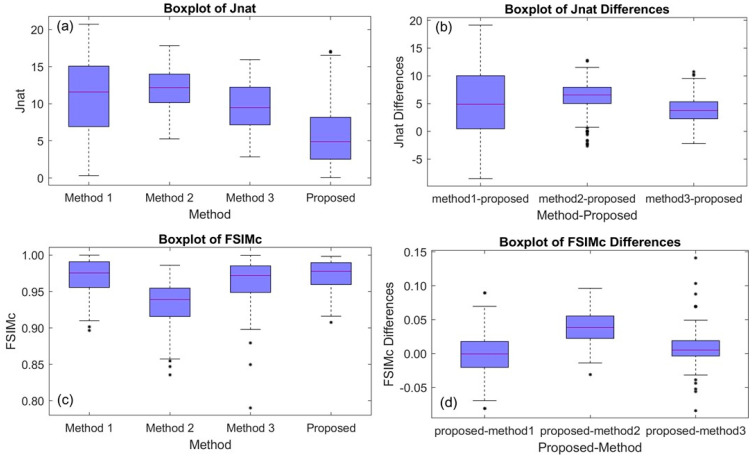
Boxplots for deuteranopia case considering the McGill’s Flowers and Fruits data sets (in total 195 images): (**a**) the Jnat values (see Table 6), (**b**) Jnat differences between the three competing methods and the proposed method (see Table 7), (**c**) FSIMc values (see Table 6), and (**d**) FSIMc differences between the three competing methods and the proposed method (see Table 7).

Table 6 and Figure 8a summarize the Jnat results for deuteranopia. There were statistically significant differences in median Jnat between the four methods. The overall *p*-value was <0.001 (based on the Friedman test).

Table 7 and Figure 8b provide a summary of the differences in Jnat between our proposed method and the other three methods (competing method value–proposed method value). The median difference in Jnat between our method and any competing method was statistically significantly greater than zero. The *p*-values are Bonferroni adjusted and based on the Wilcoxon signed rank testing procedure. Our proposed method was superior to the other three methods.

Table 6 and Figure 8c summarize the FSIMc results for deuteranopia. There were statistically significant differences in median FSIMc between the four methods (*p*-value < 0.001).

Finally, Table 7 and Figure 8d provide a summary of the differences in FSIMc (proposed method value–competing method value). The *p*-values are Bonferroni adjusted and based on the Wilcoxon signed rank testing procedure. We conclude that our proposed method was superior to Method 2 and Method 3. On the other hand, the median difference in FSIMc between our method and Method 1 was not statistically significant.

In summary, there were statistically significant differences between the four methods for all four CVD types by index combinations. Follow up testing revealed that: (a) our proposed method performed better than the other three methods in terms of Jnat, for both protanopia and deuteranopia, (b) our proposed method performed better than Method 2 and Method 3 in terms of FSIMc, for both protanopia and deuteranopia, (c) there was no difference in performance in terms of FSIMc between our proposed method and Method 1, either for protanopia or deuteranopia.

### 4.2. Qualitative Evaluation

**Figure 9:**
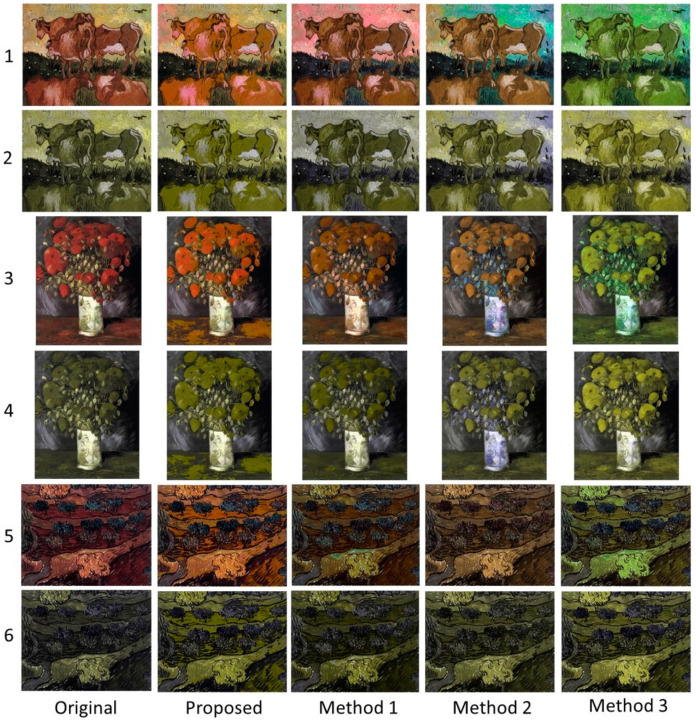
Qualitative comparison for protanopia using Paintings (**1**,**3**, and **4**). The original and the recolored paintings are given in rows (**1**,**3**, and **5**), while their respective protanope simulations are given in rows (**2**,**4**, and **6**).

In this section visual comparison is conducted considering the paintings in Figure 5. Figure 9 illustrates the results for the case of protanopia using Paintings 1, 3, and 4. Considering Painting 1 (rows 1 and 2), the proposed recolored image clearly enhances the contrast of the depicted cows. In addition, it softly modifies the background and the grass, maintaining the overall painting’s naturalness. Those properties are passed in the dichromat simulation of the recolored painting, also. On the other hand, Methods 1 and 2 enhance the contrast by mainly modifying the background and the grass. However, this strategy imposes an apparent negative effect on the naturalness. Method 3 also changes the background but not as much as Methods 1 and 2. To enhance contrast, it chooses to use mainly pure green color for the cows resulting in an unnatural representation of the painting.

In the case of Painting 3 (rows 3 and 4), the colors used by the proposed method are closer to the original painting. Moreover, the contrast (especially in the dichromat simulated painting) is enhanced when compared to the other methods. This fact is obvious at the edge line that separates the flowers and the background. The recoloring of Method 1 seems to be more competitive to our method. In Method 2 the colors of the dichromat simulated painting look alike the colors of the respective modified painting. While the same characteristic appears in the adapted paintings obtained by Method 3, the contrast in this case provides better object discrimination than Methods 1 and 2.

In Painting 4 (rows 5 and 6) the effect of the contrast enhancement is obvious regarding the proposed method. Specifically, the method chooses colors that enable the color-blind to easily discriminate between different areas of the painting. This effect is justified by the luminance optimization process. Regarding this issue, Methods 2 and 3 seem to be more competitive to our method, while the contrast of Method 1 has not been enhanced significantly.

**Figure 10:**
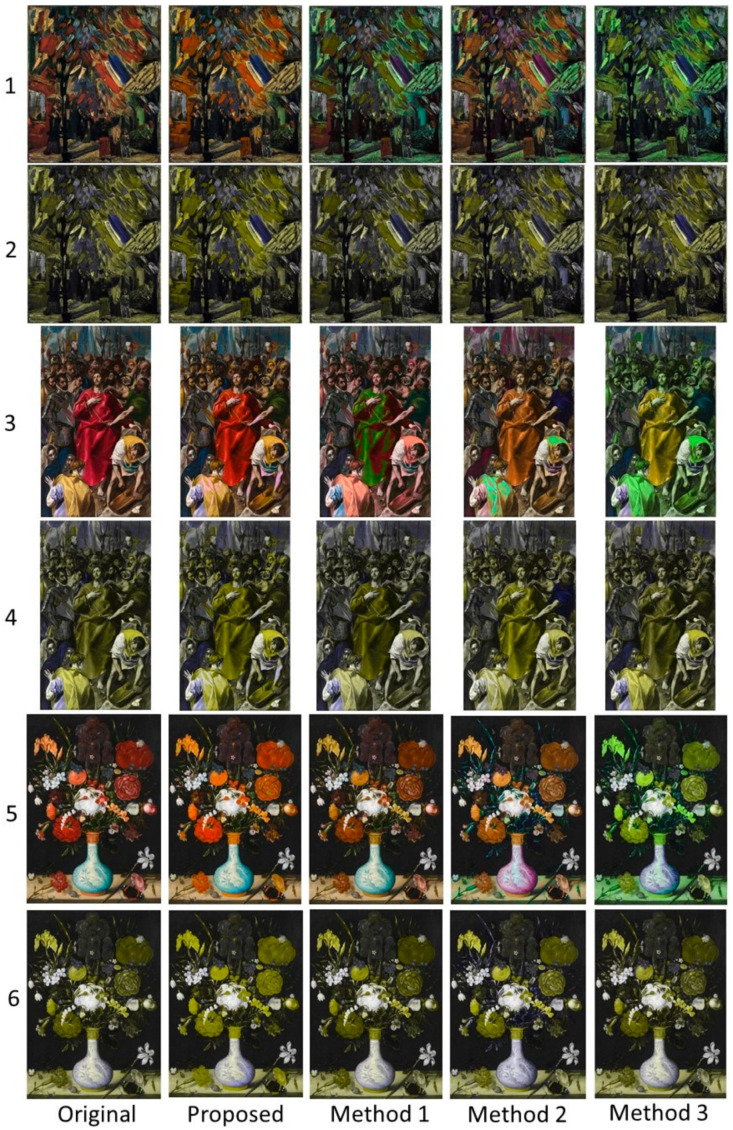
Qualitative comparison for deuteranopia using Paintings (**2**,**5**, and **6**). The original and the recolored paintings are given in rows (**1**,**3**, and **5**), while their respective deuteranope simulations are given in rows (**2**,**4**, and **6**).

Figure 10 summarizes the results for the case of deuteranopia using Paintings 2, 5, and 6. In the case of Painting 2 (rows 1 and 2), Methods 1 and 3 perform the color adaptation using mainly green color. On the other hand, Method 2 is slightly diversified from the above two methods. As far as the proposed method is concerned, the adapted colors are closer to the original colors, retaining the painting’s aesthetics and therefore its naturalness. Note that in the simulated painting of the proposed method the different areas of the paintings are clearly distinguishable one from another.

In the case of Painting 5 (rows 3 and 4) the proposed algorithm obtains the best tradeoff between naturalness and contrast when compared to the rest of the methods. For example, the color of the cloak in our recolored painting resembles the color of the original image, while the dichromat simulated painting obtains more pleasant contrast enhancement than the other three methods.

Finally, in the case of Painting 6 (rows 5 and 6), the results support the above-mentioned remarks. Clearly the recolored painting obtained by the proposed method chooses more pleasant colors retaining the overall image aesthetics. Moreover, in the dichromat simulated image, the contrast is effectively enhanced providing better discrimination abilities for the color-blind.

### 4.3. Subjective Evaluation

The four methods were evaluated in terms of pairwise comparisons performed by 28 volunteers. The responses of the volunteers were completely anonymous and no personally identifiable information was captured. Each volunteer was subjected to the well-known Ishihara test [43], which identifies if a person is protanope, deuteranope, or has normal color vison. The test suggested that eight are normal color viewers, two protanopes, seven protanomalous trichromats, three deuteranopes, and eight deuteranomalous trichromats.

Color confusions between protanopes and protanomalous trichromats are qualitatively similar, while the same holds between the deuteranopes and deuteranomalous trichromats [3,44]. This remark justifies term “protan” for protanopia/protanomaly and “deutan” for deuteranopia/deuteranomaly [3,44]. Based on this remark, three groups were identified: (a) Group1 including eight normal color vision viewers; (b) Group 2 including nine protan viewers; (c) Group 3 including 11 deutan viewers. The tested images were the six paintings reported in Figure 5. To carry out the evaluation, the subsequent three questions were used. Q1: “Which image looks more similar to the original one?” Q2: “Which image has the most pleasant contrast?” Q3: “What is your overall preference?” The first question refers to the naturalness, the second to the contrast, and the third to the overall appearance of the recolored images.

For Group 1 two experiments took place. In the first experiment, called experiment 1, viewers were asked to indicate their binary preferences for the above-mentioned questions regarding the protanopia (protan case) recolored images. Given the six images and the eight viewers belonging to Group 1, the comparison between a specific pair of methods was conducted using 48 binary choices for each pairwise comparison. Since there were six pairwise cases, the number of binary choices, called preference scores, between all pairs of algorithms was equal to 288 per question. In the second experiment, called experiment 2, the viewers did the same procedure regarding the deuteranopia (deutan case) recolored images, obtaining again 288 preference samples per question.

For Group 2 and Group 3, the first question Q1 was discarded. The reason behind this choice relies on the fact that when protans and deutans look at the original image they do not perceive it correctly, and any attempt to judge whether the recolored image is similar or not to the original will fail. Therefore, for Groups 2 and 3 only the questions Q2 and Q3 were considered for the protanopia and deuteranopia recolored paintings, respectively. Given the six images and the nine viewers belonging to Group 2, the comparison for a specific pair of algorithms was conducted using 54 binary choices. Since there were six pairwise cases, the total number of binary preferences between all pairs of algorithms was equal to 324 per question. Following the same analysis for the Group 3, which included 11 participants, we obtained a set of 396 binary choices for each question.

The pairwise-comparison data were analyzed by the Thurstone’s law of comparative judgement, case V [45]. Case V of Thurstone’s comparative judgment law is a classical tool to perform ranking of items based on subjective choices of individuals by measuring the individuals’ preference orderings for some stimuli taken from a set of discrete binary choices. In his seminal paper [45], Thurstone proposed a solution in calculating the average preference scores for each item, which Mosteller later showed was the solution to a least squares’ optimization problem [46]. In this paper, the items correspond to the four methods, and the Thurstone–Mosteller fitting model is used to estimate the average preference scores.

**Figure 11:**
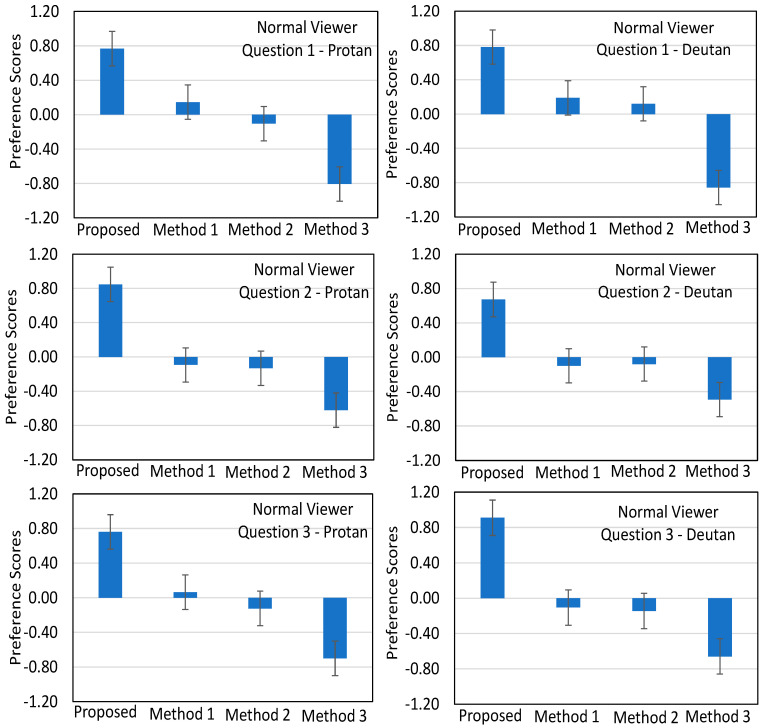
Average preference scores (blue column bars) and the corresponding 95% confidence intervals (error bars) of the pairwise comparisons for the participants belonging to Group 1: (**left column**) reports the results for experiment 1 (recolored paintings for protanopia) for the three questions, and (**right column**) reports the results for the experiment 2 (recolored paintings for deuteranopia) for the three questions.

**Figure 12:**
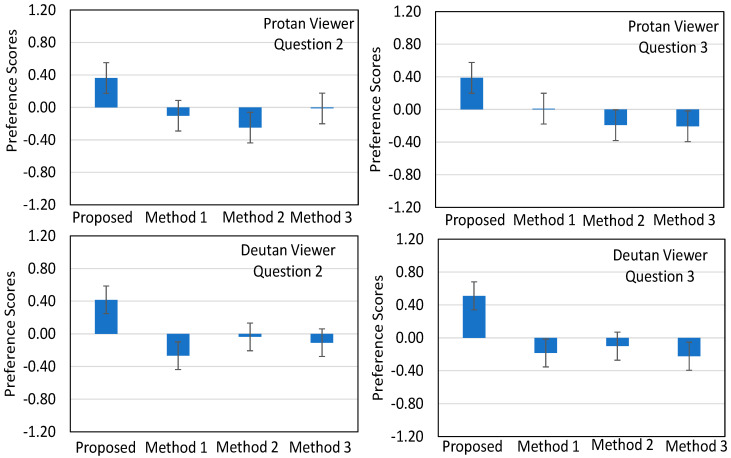
Average preference scores (blue column bars) and the corresponding 95% confidence intervals (error bars) of the pairwise comparisons for the participants belonging to Group 2 and 3: (**first raw**) reports the results for the protan viewers regarding the protanopia recolored images and the questions 2 and 3, and (**second raw**) reports the results for the deutan viewers regarding the deuteranopia recolored paintings and the questions 2 and 3.

The results from the above analysis are depicted in Figure 11 for the Group 1, and in Figure 12 for the Groups 2 and 3.

Figure 11 illustrates the average preference scores and the corresponding 95% confidence intervals of the pairwise comparisons between the four methods with respect to the normal color vision participants regarding the three aforementioned questions Q1, Q2, and Q3. Recalling that Q1 refers to the naturalness, Q2 to the contrast, and Q3 to the overall appearance of the recolored paintings, we can easily point out the following observations.

First, the analysis indicates that the normal color vision viewers clearly preferred the proposed method for all questions and cases of recolored paintings (protan and deutan). In particular, the participants’ preference to the proposed method becomes more evident in Question 2—Protan, Question 2—Deutan, and Question 3—Deutan cases. Second, the most competitive method to our proposed one is Method 1 (Huang et al. [25]). Indeed, apart from the case Question 2—Deutan, that method outperformed the other two. Third, Method 2 (Wong and Bishop [27]) performs better than Method 3 (Ching and Sabudin [28]).

Figure 12 illustrates the average preference scores and the corresponding 95% confidence intervals of the pairwise comparisons between the four methods with respect to Group 2 and Group 3, regarding Questions 2 and 3. The analysis illustrated in this figure directly indicates the superiority of the proposed method, supporting the results reported in Figure 11. However, the difference between the proposed and the other three methods has been clearly reduced for both the protans and deutans when compared to the results in Figure 11. As far as the other three methods are concerned, Method 2 outperforms the others in two cases of deutan viewers (second raw in Figure 12), while each one of Method 1 and Method 3 in only one, respectively.

In summary, the results of the subjective evaluations show that the proposed method improves the visual details, enhances the contrast, and provides a pleasant appearance for normal color vision viewers as well as for protans and deutans.

## 5. Discussion and Conclusions

This contribution presents a novel method to modify color images for protanopia and deuteranopia color vision deficiencies. The method consists of four main modules, which are applied in sequence. The first module concerns the color segmentation of the input image and the generation of a number of key colors that might be confusing or non-confusing for the color-blind. The second module maps the key colors onto CIE 1931 chromaticity diagram, where a sophisticated mechanism removes confusing key colors that lie on the same confusion line to different confusion lines so that they can be discriminated by the color-blind. Specifically, each confusing key color is translated to its closest non-occupied confusion line. Next, the above modified key colors are further optimized in the third module through a regularized objective function. Finally, the fourth module obtains the recolored image by adapting the pixels of the input image that correspond to confusing key colors using a color transfer technique.

The implementation of the proposed method is carried out by considering two basic criteria. First, the preservation of the natural appearance of the recolored image in relation to the original input image. Second the contrast enhancement between different image areas. The former is related to the preservation of the recolored image aesthetics as perceived by a normal color vision viewer. The latter refers to the discrimination of image areas that include colors confused by the color-blind.

The above-mentioned criteria are implemented throughout the whole method. To justify that conclusion the following remarks are pointed out. First, the image pixels that are recolored are those that correspond to the confusing key colors. Thus, the part of the input image that corresponds to non-confusing colors remains unchanged; a fact that favors the naturalness criterion. Second, in the key color translation process, the key colors that lie on the same confusion line are removed to different confusion lines. This strategy enhances the contrast because colors that were initially confused can now be discriminated by the color-blind. Moreover, since each confusing key color is moved to its closest non-occupied confusion line, the recolored key colors will be close to the original ones, preserving the naturalness criterion. Finally, the optimization procedure presented in the third module uniformly takes into account both criteria and finally obtains an optimal recoloring of the input image.

For a full validation of the proposed method, extensive experimental studies were conducted. The validation demonstrated the improved performance compared to other three recoloring methodologies. By way of the next steps, future research efforts could be made in the following directions. First, in extending the methodology to cope with the tritanopia and anomalous trichromacy defects. Second, in developing more effective machine learning approaches in the recoloring process. Third, in implementing more sophisticated optimization procedures to maintain an optimal tradeoff between naturalness preservation and contrast enhancement.

## Figures and Tables

**Table 1:** Parameter setting for the proposed method.

Module 1		Modules 2 and 3		Differential Evolution	
Parameter	Value	Parameter	Value	Parameter	Value
ρ	10	$Q_{D}$ (Protanopia)	17	q	20
δ	25	$Q_{D}$ (Deuteranopia)	15	$F_{R}$	0.8
$n_{A}$	5	γ	5	$C_{R}$	0.6
$n_{B}$	5	λ	0.2	$t_{\max}$	100

**Table 2:** Naturalness index (Jnat) values obtained by the four methods for protanopia and deuteranopia considering the paintings reported in Figure 5.

	Protanopia				Deuteranopia			
Painting	Proposed	Method 1	Method 2	Method 3	Proposed	Method 1	Method 2	Method 3
1	4.8324	9.9372	13.2600	12.0398	7.4648	13.6121	13.2600	9.6462
2	2.2735	7.7299	8.3201	8.4141	2.4797	9.1270	8.3201	5.6994
3	4.4311	6.5477	9.3297	9.5566	2.1356	4.6454	9.3297	6.9328
4	4.2495	6.5726	11.8014	11.7561	4.1392	4.5544	11.8014	9.4815
5	3.7393	7.5320	8.7266	10.8788	3.9259	7.9379	8.7266	7.5273
6	2.6914	2.4915	6.7193	6.4676	2.6676	1.5740	6.7193	4.7121

**Table 3:** Feature similarity index (FSIMc) values obtained by the four methods for protanopia and deuteranopia considering the paintings reported in Figure 5.

	Protanopia				Deuteranopia			
Painting	Proposed	Method 1	Method 2	Method 3	Proposed	Method 1	Method 2	Method 3
1	0.9386	0.9828	0.9654	0.9342	0.9443	0.9430	0.9654	0.9752
2	0.9939	0.9832	0.9663	0.9518	0.9690	0.9936	0.9663	0.9922
3	0.9891	0.9735	0.9254	0.9099	0.9922	0.9831	0.9254	0.9788
4	0.9454	0.9747	0.9647	0.9141	0.9212	0.9778	0.9647	0.9707
5	0.9911	0.9882	0.9676	0.9545	0.9907	0.9854	0.9676	0.9828
6	0.9895	0.9917	0.9631	0.9423	0.9953	0.9760	0.9631	0.9842

**Table 4:** Descriptive Statistics of the Jnat and FSIMc indices for the protanopia case considering the McGill’s Flowers and Fruits data sets (in total 195 images).

Method	Min	1st Quartile (Q1)	Median	3rd Quartile (Q3)	Max
**Jnat**					
Method 1	0.994	8.579	12.881	16.082	22.042
Method 2	5.262	10.149	12.181	14.003	17.825
Method 3	6.494	11.649	13.277	14.783	20.177
Proposed	0.036	2.297	4.802	8.208	17.376
**FSIMc**					
Method 1	0.897	0.955	0.972	0.986	0.999
Method 2	0.836	0.916	0.939	0.955	0.986
Method 3	0.756	0.889	0.926	0.947	0.988
Proposed	0.885	0.959	0.973	0.986	1.000

**Table 5:** Descriptive statistics of the observed Jnat and FSIMc differences between the three competing methods and the proposed method for the protanopia case considering the McGill’s Flowers and Fruits data sets (in total 195 images).

Method	Min	Q1	Median	Q3	Max	95% CIs for Medians (Bonferroni adj.)	*p*-Value (Bonferroni adj.)
	**Jnat Differences (Method–Proposed)**						
Method 1	−9.887	3.214	6.579	10.987	18.659	(5.736, 8.035)	<0.015
Method 2	−3.085	4.611	7.020	8.435	13.017	(6.016, 7.400)	<0.015
Method 3	−1.251	4.912	7.475	10.254	14.898	(6.810, 8.208)	<0.015
	**FSIMc Differences (Proposed–Method)**						
Method 1	−0.078	−0.012	0.002	0.015	0.072	(−0.001,0.006)	0.603
Method 2	−0.046	0.022	0.037	0.053	0.118	(0.033, 0.040)	<0.015
Method 3	−0.041	0.027	0.049	0.076	0.198	(0.042, 0.058)	<0.015

**Table 6:** Descriptive statistics of the Jnat and FSIMc indices for the deuteranopia case considering the McGill’s Flowers and Fruits data sets (in total 195 images).

Method	Min	1st Quartile (Q1)	Median	3rd Quartile (Q3)	Max
**Jnat**					
Method 1	0.312	6.901	11.590	15.072	20.727
Method 2	5.262	10.149	12.181	14.003	17.825
Method 3	2.842	7.155	9.485	12.239	15.946
Proposed	0.045	2.523	4.890	8.197	17.076
**FSIMc**					
Method 1	0.897	0.956	0.976	0.910	1.000
Method 2	0.836	0.916	0.939	0.955	0.986
Method 3	0.790	0.949	0.972	0.985	1.000
Proposed	0.908	0.960	0.978	0.990	0.998

**Table 7:** Descriptive statistics of the observed Jnat and FSIMc differences between the three competing methods and the proposed method for the deuteranopia case considering the McGill’s Flowers and Fruits data sets (in total 195 images).

Method	Min	Q1	Median	Q3	Max	95% CIs for Medians (Bonferroni adj.)	*p*-Value (Bonferroni adj.)
	**Jnat Differences (Method–Proposed)**						
Method 1	−8.516	0.460	4.880	10.072	19.144	(3.569, 7.572)	<0.015
Method 2	−2.655	5.022	6.558	7.945	12.800	(5.866, 7.151)	<0.015
Method 3	−2.209	2.259	3.768	5.335	10.739	(3.223, 4.345)	<0.015
	**FSIMc Differences (Proposed–Method)**						
Method 1	−0.081	−0.020	−0.001	0.018	0.090	(−0.005, 0.006)	1.000
Method 2	−0.031	0.022	0.038	0.056	0.096	(0.033, 0.044)	<0.015
Method 3	−0.084	−0.004	0.005	0.019	0.141	(0.001, 0.009)	<0.015

## Data Availability

Not applicable.

## Appendix A

In this Appendix, a brief description of the form of the FSIMc index is given. Further details can be found in reference [42]. The analysis involves the input image $P=\left[p_{t_{1}t_{2}}\right]$ and its recolored image $P_{rec}=\left[p_{t_{1}t_{2},rec}\right]$, $\left(1\leq t_{1}\leq N;\;\;1\leq t_{2}\leq M\right)$.

To obtain the FSIMc index the above two images are transformed into the *YIQ* color space. The transformation of $p_{t_{1}t_{2}}$ is $p_{t_{1}t_{2}}^{YIQ}={\left[p_{t_{1}t_{2}}^{Y}\;\;p_{t_{1}t_{2}}^{I}\;\;p_{t_{1}t_{2}}^{Q}\right]}^{T}$. Relationally, the transformation of $p_{t_{1}t_{2},rec}$ is $p_{t_{1}t_{2},rec}^{YIQ}={\left[p_{t_{1}t_{2},rec}^{Y}\;\;p_{t_{1}t_{2},rec}^{I}\;\;p_{t_{1}t_{2},rec}^{Q}\right]}^{T}$. Only for the luminance channel *Y*, for each of the above pixels, we calculate the phase congruencies (*PC*), which are denoted as $PC(p_{t_{1}t_{2}}^{Y})$, $PC(p_{t_{1}t_{2},rec}^{Y})$. In addition, only for the luminance channel, we calculate the respective image gradient magnitudes (*G*), which are denoted as $G(p_{t_{1}t_{2}}^{Y})$, $G(p_{t_{1}t_{2},rec}^{Y})$.

The maximum value between $PC(p_{t_{1}t_{2}}^{Y})$, $PC(p_{t_{1}t_{2},rec}^{Y})$ is

(A1) $$PC_{\max,\;t_{1}t_{2}}=\max\left\{PC(p_{t_{1}t_{2}}^{Y}),\;\;PC(p_{t_{1}t_{2},rec}^{Y})\right\}$$

Next, the following similarity measures are estimated,

(A2) $$S_{PC,\;t_{1}t_{2}}=\frac{2\;PC(p_{t_{1}t_{2}}^{Y})\;PC(p_{t_{1}t_{2},rec}^{Y})\;+\theta_{1}}{{\left(PC(p_{t_{1}t_{2}}^{Y})\right)}^{2}+{\left(PC(p_{t_{1}t_{2},rec}^{Y})\right)}^{2}+\theta_{1}\;\;}\;$$

(A3) $$S_{G,\;t_{1}t_{2}}=\frac{2\;G(p_{t_{1}t_{2}}^{Y})\;G(p_{t_{1}t_{2},rec}^{Y})\;+\theta_{2}}{{\left(G(p_{t_{1}t_{2}}^{Y})\right)}^{2}+{\left(G(p_{t_{1}t_{2},rec}^{Y})\right)}^{2}+\theta_{2}\;\;}\;$$

Relationally, for the chromaticity channels *I* and *Q* the subsequent similarity measures are estimated,

(A4) $$S_{I,\;t_{1}t_{2}}=\frac{2\;\;p_{t_{1}t_{2}}^{I}\;p_{t_{1}t_{2},rec}^{I}\;+\theta_{3}}{{\left(p_{t_{1}t_{2}}^{I}\right)}^{2}+{\left(p_{t_{1}t_{2},rec}^{I}\right)}^{2}+\theta_{3}\;\;}\;$$

(A5) $$S_{Q,\;t_{1}t_{2}}=\frac{2\;\;p_{t_{1}t_{2}}^{Q}\;p_{t_{1}t_{2},rec}^{Q}\;+\theta_{4}}{{\left(p_{t_{1}t_{2}}^{Q}\right)}^{2}+{\left(p_{t_{1}t_{2},rec}^{Q}\right)}^{2}+\theta_{4}\;\;}\;$$

To this end, the FSIMc index is defined as follows,

(A6) $$\mathrm{FSIMc}=\frac{\sum_{t_{1},\;t_{2}}S_{PC,\;t_{1}t_{2}}S_{G,\;t_{1}t_{2}}\;{\left(S_{I,\;t_{1}t_{2}}\;S_{Q,\;t_{1}t_{2}}\right)}^{\eta }\;PC_{\max,\;t_{1}t_{2}}}{\sum_{t_{1},\;t_{2}}PC_{\max,\;t_{1}t_{2}}}$$

The detailed calculation procedure of the phase congruencies and image gradient magnitudes as well the appropriate values for the parameters $\theta_{1},\;\;\theta_{2},\;\;\theta_{3},\;\;\theta_{4},$ and η are given in reference [42].
